# Dermatology

**Published:** 1897-02-06

**Authors:** 


### DERMATOLOGY.

(*Continued from page* 299.)

**Parasitic Diseases.**—Colcott, Fox, and Blaxall,[11] in an inquiry into the plurality of fungi causing ringworm in human beings, as met with in London, have followed the methods adopted by Sabouraud in Paris. They found no difficulty in recognising examples of microsporon, and thrichophyton endothrix, and ectothrix, since the differences were so well marked that in the majority of cases a clinical and microscopical examination was conclusive. They found that both the endothrix and the ectothrix were comparatively rare; but the microsporon caused something like 80 to 90 per cent. of ringworms met with in London; with regard to age, it attacked principally children under six years. They disagree with Sabouraud in considering that the scalp is implicated only secondarily to the hair, since if the skin of a ringworm patch be examined in the early stages, there will always be found a quantity of mycelial feltwork even before the hairs are appreciably affected. They found less difficulty in recognising the endothrix, both clinically and microscopically, than any other of the fungi, but no example of the peladoid form had come before them. Clinically, they suspected the nature of the majority of the cases, because of the occurrence of isolated or disseminated stumps, or of finger-nailed sized areas with three or four stumps, the hairs of which were certainly very fragile, and broke off short. With regard to ectothrix, the clinical characters were usually of an inflammatory and sometimes pustular type; the hairs are not disintegrated so rapidly as by the microsporon, and sometimes nothing but apparently healthy hairs are seen traversing the scalp. They found in cases of kerion a small-spored fungus which cannot be distinguished from the microsporon. In cases of tinea sycosis the fungi are beautiful examples of ectothrix of large kinds. In their cultivation experiments, they lay special stress upon the importance of removing the normal parts of the hair from the specimen, as otherwise there would certainly ensue a copious growth of common mould which would obscure the other fungus. They recommend a medium made by steaming powdered potato in water, straining, and then adding 1·5 per cent. of agar-agar and 3 per cent. of pure maltose. They were able to perceive, by the use of Klatsch specimens, that the aërial fructification of the microspora of the endothrix, of the ectothrix, and of the cat group are formed on a similar plan. They found chlamydospores invariably in microspora, frequent in ectothrixes, and absent in the endothrixes; they therefore think that we are justified in considering that all this species belong to one and the same family. They consider that the differences which they have observed to those found by Sabouraud were due not to dissimilarity of organisms, but to different methods of examination.

Krösing[12] has also investigated the Thrichophyton fungus, and does not agree with Sabouraud's division into large and small spores. The culture media, which are preferable, are made of potatos, and the mycelial threads are separated from the hairs by energetic agitation, preferably with water. He considers that superficial and deep affections (viz., sycosis and thrichophytia circinata) may be caused by the same variety, and he further states that suppuration may be produced by the thrichophyton alone without the presence of other organisms.

Duhring and Hartzell[13] report upon the investigations recently made by Ulman, confirming those of

Sabouraud, upon the etiology of sycosis; in all the cases there was an ectothrical form of thrichophytosis, in which the elements of the fungus remain in the hair-sheath without entering into the substance of the hair. Differences in the extent and in the intensity of the affection are explained by different degrees of virulence of the organism, sensitiveness of the skin, chemical reaction of the sweat, and neglect of the skin. Clinically some cases resemble impetigo contagiosa, and can only be differentiated by the presence of a trichophyton under the microscope. The suppuration of the hair follicle leads not only to the falling out of the hair, but to destruction of the sebaceous glands belonging to the follicle. This extensive suppuration in the follicular tissues is explained by the presence of a toxin formed by the fungus.

Freeman[14] lays special stress upon the influence of damp surroundings in preventing the cure of ringworm. He therefore suggests that the patient should be removed to a high-standing sanitary house. With regard to general treatment, he gives cod-liver oil to children of the fair-haired, scrofulous type, and small doses of Fowler's solution to delicate anæmic children, and orders them upon all possible occasions to have the scalp freely exposed to the influence of the sun and air. He commences local treatment by scrubbing with carbolic or coal-tar soap, and rubbing in turpentine till the child cries out. Some antiseptic ointment is then rubbed in several times daily. When only a few diseased hairs are left they are epilated, and the follicles destroyed with a red-hot wire.

[11] B. Journ. Derm., July, 1896, p. 241; ditto, Aug., p. 292; ditto, Sept., p. 377. [12] Int. Med. Mag., Aug. 1, 1896, p. 486; B. Journ. Derm., Oct., 1896, p. 400. [13] Am. Journ. Med. Sci., Oct., 1896, p. 488. [14] Lancet, Oct. 31, 1896, p. 1,219.

